# Moesin contributes to heat shock gene response through direct binding to the Med15 subunit of the Mediator complex in the nucleus

**DOI:** 10.1098/rsob.240110

**Published:** 2024-10-02

**Authors:** Ildikó Kristó, Zoltán Kovács, Anikó Szabó, Péter Borkúti, Alexandra Gráf, Ádám Tamás Sánta, Aladár Pettkó-Szandtner, Edit Ábrahám, Viktor Honti, Zoltán Lipinszki, Péter Vilmos

**Affiliations:** ^1^ Institute of Genetics, HUN-REN Biological Research Centre, Szeged, Hungary; ^2^ HCEMM-BRC Mutagenesis and Carcinogenesis Research Group, Institute of Genetics, HUN-REN Biological Research Centre, Szeged, Hungary; ^3^ Doctoral School of Biology, Faculty of Science and Informatics, University of Szeged, Szeged, Hungary; ^4^ Delta Bio 2000 Ltd., Szeged 6726, Hungary; ^5^ Proteomics Laboratory, HUN-REN Biological Research Centre, Szeged, Hungary; ^6^ MTA SZBK Lendület Laboratory of Cell Cycle Regulation, Synthetic and Systems Biology Unit, Institute of Biochemistry, HUN-REN Biological Research Centre, Szeged, Hungary; ^7^ National Laboratory for Biotechnology, Institute of Genetics, HUN-REN Biological Research Centre, Szeged, Hungary

**Keywords:** moesin, actin, nucleus, gene expression, mediator, heat shock

## Abstract

The members of the evolutionary conserved actin-binding Ezrin, Radixin and Moesin (ERM) protein family are involved in numerous key cellular processes in the cytoplasm. In the last decades, ERM proteins, like actin and other cytoskeletal components, have also been shown to be functional components of the nucleus; however, the molecular mechanism behind their nuclear activities remained unclear. Therefore, our primary aim was to identify the nuclear protein interactome of the single *Drosophila* ERM protein, Moesin. We demonstrate that Moesin directly interacts with the Mediator complex through direct binding to its Med15 subunit, and the presence of Moesin at the regulatory regions of the *Hsp70Ab* heat shock gene was found to be Med15-dependent. Both Moesin and Med15 bind to heat shock factor (Hsf), and they are required for proper *Hsp* gene expression under physiological conditions. Moreover, we confirmed that Moesin, Med15 and Hsf are able to bind the monomeric form of actin and together they form a complex in the nucleus. These results elucidate a mechanism by which ERMs function within the nucleus. Finally, we present the direct interaction of the human orthologues of *Drosophila* Moesin and Med15, which highlights the evolutionary significance of our finding.

## 1. Introduction

Moesin belongs to the evolutionarily highly conserved actin-binding Ezrin, Radixin and Moesin (ERM) protein family of vertebrates. The main function of ERM proteins is to mediate the attachment of the cortical actin cytoskeleton to transmembrane proteins with the help of filamentous actin (F-actin). Therefore, ERMs act as key players in numerous basic cellular activities, such as cell movement and shaping, the formation of microvilli, or the regulation of the activity of signal transduction pathways [1–5]. All ERM proteins share a very similar structure: the protein- and lipid-binding FERM domain occupies the N-terminal region, followed by a flexible middle domain and a C-terminal F-actin binding domain. Interestingly, ERM proteins and several other FERM domain-containing proteins were also found to be present in the cell nucleus (reviewed in [6]), which is consistent with the fact that many cytoskeletal components are also located in the nucleus [7]. Recently, the biological significance of nuclear ERMs has also become clear, as it has been shown that *Drosophila* Moesin participates in mRNA export [8] and is also required for the proper expression of *Hsp* genes [9].

ERM proteins use their FERM domain, which acts as a switchable interaction hub, to bind numerous different protein partners to ensure their widespread functions in the cytoplasm. Therefore, based on the already described nuclear functions of Moesin, we can assume that they should probably have a similarly large number of interactors in the nucleus as well. The most important binding partner of ERMs, actin, is also present in the nucleus and is known to be part of almost all major nuclear complexes and is therefore involved in many nuclear processes, such as DNA repair, replication, chromatin rearrangements, transcription, splicing and mRNA export [10–14]. We hypothesize that, like actin, with its diverse tasks in the nucleus, ERMs may function also in a variety of nuclear processes. However, it is still unclear whether ERMs and actin can work together in the nucleus, since the main form of nuclear actin is the monomeric (G-actin) and not the filamentous form, which is the favourable state for ERM binding. However, the binding of G-actin to ERM proteins is indicated by previous literature data [15] and is also suggested by our results presented in this study, which open a new field in the molecular function of ERMs.

In terms of its composition and structure, Mediator is a highly conserved multi-subunit complex in eukaryotes, which performs very diverse functions in the nucleus. The complex can be divided into four main, functionally distinct domains, the head, middle and tail domains, as well as a reversibly associated, regulatory cyclin-dependent kinase (CDK) domain. One of the main tasks of Mediator is to transmit the signals from the transcription regulators bound to the regulatory regions to the transcription machinery, thus facilitating the formation of the pre-initiation complex on the promoter of almost all enhancer-regulated genes. Furthermore, it also plays an essential role in transcription elongation, termination and post-transcriptional steps of gene expression, such as mRNA export, and coordinates these events as well [16–19]. In addition, Mediator is also involved in gene regulation induced by extracellular stress, such as heat shock or starvation [20–23]. The fundamental biological role of the Mediator complex is also supported by the fact that numerous human diseases are already associated with mutations or altered expression of certain subunits [24,25]. In accordance with its multiple roles in the cells, the complex exhibits a dynamic protein composition (25 subunits in budding yeast and up to 30 subunits in human). As we previously demonstrated, the sole *Drosophila* ERM protein, Moesin, is involved in mRNA export [8] and heat shock gene expression [9] in the nucleus; therefore, we hypothesized that the activity of the Mediator complex could be a possible way through which Moesin performs its functions in the nucleus. In this study, we aimed to identify the nuclear protein interactome of Moesin in order to explore and better understand its molecular role in the nucleus and to identify its possible new nuclear functions.

## 2. Results

### 2.1. Moesin interacts with proteins involved in diverse nuclear functions

**Figure 1.**
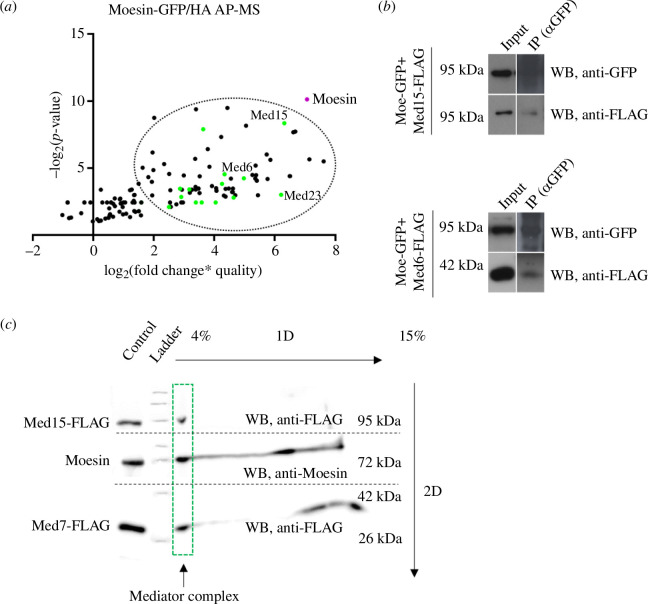
Moesin interacts with the Mediator complex. (*a*) LC-MS/MS analysis of immunoprecipitated proteins from nuclear protein fractions of S2R+ cells expressing either Moesin-GFP or Moesin-HA, respectively. Moesin immunoprecipitation was done by anti-GFP or anti-HA antibody-conjugated beads. Dots represent the first 150 proteins with the highest score calculated from six independent experiments (three of Moesin-GFP and three of Moesin-HA). Almost all Mediator complex members (green dots) were among the top hits (encircled). AP-MS, affinity purification-coupled to mass spectrometry. (*b*) Co-immunoprecipitation with anti-GFP antibody on protein lysate of S2R+ cells co-expressing Moesin-GFP and Med15-FLAG or Med6-FLAG, respectively. IP – immunoprecipitation. WB, western blot. (*c*) Two-dimensional BN-PAGE performed on nuclear fraction of S2R+ cells co-expressing Med15-FLAG and Med7-FLAG. Green rectangle marks the lane corresponding to the Mediator complex. Endogenous Moesin was detected in the same lane with Med15-FLAG and Med7-FLAG indicating that they are in the same nuclear complex. Broken line indicates the margins of western blot membranes developed with anti-FLAG or anti-Moe antibody. 1D, one-dimensional; 2D, two-dimensional; WB, western blot.

The most important interaction partner of Moesin is actin, which has fundamental roles in various nuclear processes, and accordingly, the presence of actin has already been confirmed in numerous nuclear complexes, and in some cases, the molecular mechanism behind the activity of nuclear actin has also been revealed [26,27]. Due to actin’s widespread and important functions in the nucleus, and the fact, that Moesin is also involved in nuclear activities [8,9], we hypothesized that Moesin might also play multiple roles in the nucleus in association with actin, just as is the case in the cytoplasm [28,29]. To explore the molecular mechanism(s) behind Moesin’s function in the nucleus, we performed an affinity purification-coupled to mass spectrometry (AP-MS) analysis on nuclear protein fraction of cultured *Drosophila* S2R+ cells. Using transiently transfected cells expressing either GFP- or HA-tagged Moesin, we isolated nuclear and cytoplasmic protein fractions and performed immunoprecipitation (IP) by using antibodies recognizing the GFP or HA epitopes. The two types of tags were used in order to minimize non-specific binding, and the experiment was performed three times for both tags. The precipitated proteins were identified by LC-MS/MS analysis, and a score was calculated for each hit by multiplying the fold change of peptide count by the coverage percentage and the quality of the IP (figure 1*a*; electronic supplementary material, table S1). The IP performed on the cytoplasmic fraction revealed several already known non-membrane binding partners of Moesin among the first 100 hits of the list, such as Moesin itself, actin, EB1, Ter94, Rab11 and Rho1 (data not shown), thus confirming the reliability of the method.

Candidate proteins identified in the screen represented protein complexes implicated in various nuclear functions, which indicates that, similarly to actin, Moesin’s function is most likely to be related to several processes in the nucleus. Proteins already known to be involved in mRNA processing, splicing and export were present among the best hits, which nicely correlates with our previous finding that Moesin plays a role in mRNA export [8]. A significant amount of the identified proteins among the best 50 candidates participate in chromatin remodelling or in rRNA transcription, processing and in ribosome biogenesis, suggesting a possible role for Moesin in these events.

However, the most interesting and obvious finding was that almost all members of the Mediator complex were pulled down together with Moesin, which argued for a possible interaction between Moesin and the Mediator complex (figure 1*a*). We identified 24 Mediator subunits (in *Drosophila,* it comprises 30 proteins) in the first 117 proteins, where the strongest hits were Med15, Med23 and Med6 with the AP-MS rank of 7, 8 and 20, respectively (electronic supplementary material, table S1).

### 2.2. Moesin interacts with the Mediator complex

To confirm the interaction between Moesin and the Mediator complex, we co-transfected S2R+ cells with DNA constructs expressing Moesin-GFP and FLAG-tagged Mediator complex members Med15 and Med6, respectively, as they had the highest score among Mediator proteins in the AP-MS screen. Transfected cells were lysed, and co-immunoprecipitation (co-IP) was performed with anti-GFP antibody. The proper expression and localization of these proteins were validated by immunostaining (electronic supplementary material, figure S1). We found that the Med15 and Med6 subunits co-immunoprecipitated with Moesin, indicating that Moesin is in the same complex with the examined Mediator subunits (figure 1*b*). Next, we applied the blue native polyacrylamide gel electrophoresis (BN-PAGE) method that allows the separation of nuclear multiprotein complexes under native condition, which then are dissociated into their individual components in a two-dimensional gel. We selected Med15 to identify the Mediator complex, as it had the highest score in the AP-MS list among the Mediator subunits and it showed binding to Moesin in the co-IP experiments. Med7 was used as an additional marker to identify the Mediator complex on the gel due to its small molecular weight, which therefore enables the simultaneous detection of Med15, Med7 and Moesin in the same lane of the gel. We isolated nuclear protein fraction from cells co-expressing Med15 and Med7 both tagged with a FLAG tag. After the two-dimensional separation, we found that endogenous Moesin and the Mediator complex members Med15 and Med7 were detected in the same lane, further confirming that Moesin is a member of the Mediator complex in the nucleus (figure 1*c*).

### 2.3. Moesin binds Mediator complex members *in vitro*

**Figure 2.**
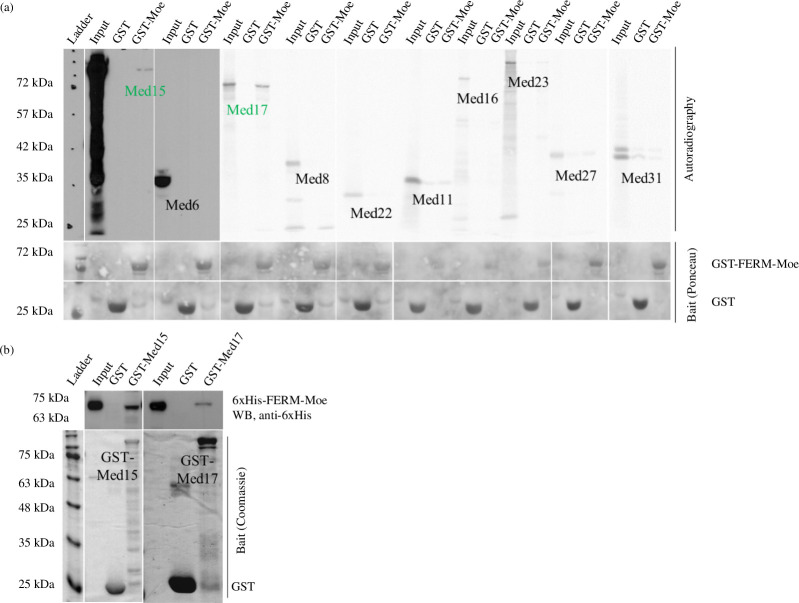
Moesin interacts with Mediator complex subunits *in vitro.* (*a*) Upper part: autoradiography images show the result of the IVTT-GST binding assay performed to investigate the interaction between GST-FERM-Moesin (bait) and Mediator complex subunits produced by IVTT reaction (preys). Lower part: Ponceau staining demonstrating the presence of GST-FERM-Moesin and GST. GST alone was used as a negative control. (*b*) Reciprocal analysis of the interactions in (*a*) with proteins purified from *Escherichia coli* bacteria. GST-Med15 and GST-Med17 (baits) pulled down the 6xHis-FERM-Moe protein (prey). GST alone was used as a negative control. WB, western blot.

To find the possible direct interaction between Moesin and the Mediator complex, we selected Mediator complex members for an *in vitro* binding screen to distinguish between direct and indirect protein interactions. Based on the AP-MS rank, the availability of full-length coding DNA sequences (CDSs) and the proper *in vitro* expression level, Med6, Med8, Med11, Med15, Med16, Med17, Med22, Med23, Med27 and Med31 proteins were investigated by *in vitro* binding assay. We generated radioactively labelled candidate proteins through a coupled *in vitro* transcription and translation (IVTT) reaction and conducted GST-IVTT binding assays using recombinant GST-FERM-Moesin protein immobilized onto affinity beads. The assay revealed direct binding of Med15 and Med17 to Moesin (figure 2*a*), whereas Med6/8/11/16/22/23/27/31 did not exhibit physical interaction with the bait protein. To further validate the interactions, we performed another *in vitro* pull-down experiment using purified recombinant proteins in a reciprocal arrangement. We immobilized GST-tagged Med15 and Med17 recombinant proteins purified from bacteria onto affinity beads and incubated them with purified, recombinant and 6xHis-tagged FERM-Moesin. We found that Moesin directly interacted with both GST-Med15 and GST-Med17 *in vitro*, confirming the results of the GST-IVTT assay (figure 2*b*).

### 2.4. Moesin co-localizes with Mediator complex members on the chromosomes

**Figure 3.**
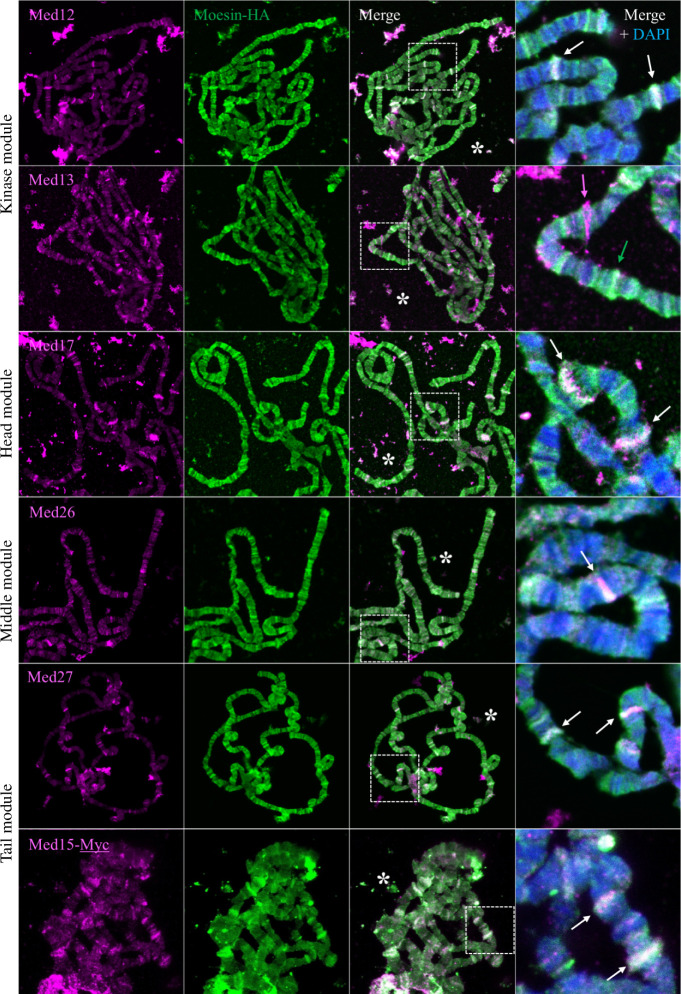
Moesin co-localizes with a subset of Mediator complex members on giant polytene chromosomes. Immunostaining of larval polytene chromosomes for the localization of HA-tagged Moesin protein and the Med12, Med13, Med17, Med26, Med27 and Med17 subunits representing the four main subdomains of the Mediator complex. Right part: higher magnification of polytene chromosomes stained for Moesin (green), Med subunits (magenta) and merged with DAPI (blue). Arrows indicate bands showing co-localization (white) or single localization (magenta or green). White stars indicate overlapping extrachromosomal co-localization.

The Mediator complex exhibits a dynamic subunit composition and a diverse structure that enables it to fulfil numerous functions. Therefore, we tested the chromosomal localization pattern of several Mediator subunits and compared them to that of Moesin. For these experiments, we selected the Med12 and Med13 (Kinase domain), Med17 (Head), Med26 (Middle), Med15 and Med27 (Tail) subunits, as antibodies specific to these proteins are available. We found that Moesin exhibits a chromosomal staining pattern very similar to that of Med12, Med15, Med17, Med26 and Med27 (figure 3 right, white arrows), albeit with different intensities. The localization of Med13 and Moesin overlapped somewhat but were largely distinct (figure 3 right, magenta and green arrows). The similar but not identical localization indicates that Moesin is most likely part of the Mediator complex, but not as a permanent component, but as a member of one or rather of a few complex forms with a specific composition and thus a function. The overlapping bands were observed always in DAPI negative bands throughout the entire chromosomes, which reveals that Moesin and the Mediator are present together in the euchromatic regions.

### 2.5. Moesin directly binds Med15 *in vivo*

To confirm the direct interaction between Moesin and the Mediator complex *in vivo*, we applied the bimolecular fluorescent complementation (BiFC) or split YFP [30] method, where we tagged Moesin and Mediator complex members with the N- or C-terminal part of the YFP protein (NYFP and CYFP, respectively). In addition to indicating the direct binding of two proteins, the BiFC method can also reveal the subcellular localization of the interaction.

**Figure 4.**
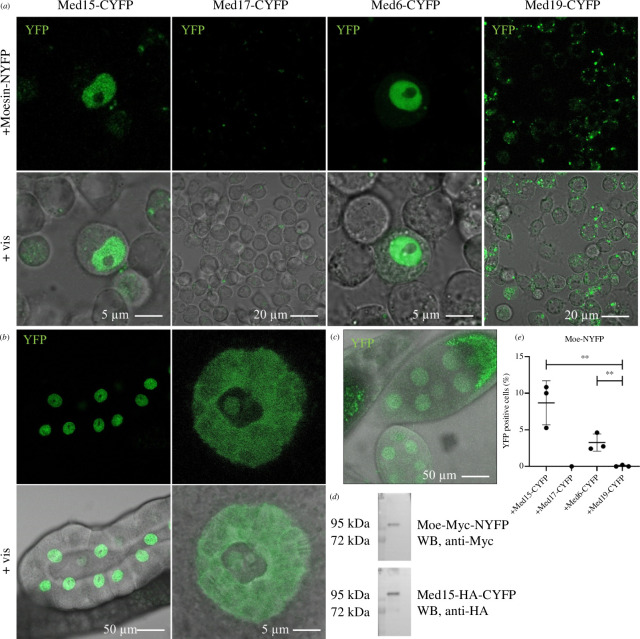
Moesin directly binds Med15 and Med6 in the nucleus *in vivo.* (*a*) Representative images of the split YFP assay revealing that Moesin directly binds to the Med15 and Med6 Mediator complex subunits in the nucleus of S2R+ cells *in vivo*. Med19 was used as a negative control. (*b,c*) The co-expression of Moe-NYFP and Med15-CYFP proteins in larval salivary glands or adult ovaries produces a strong nucleus-specific YFP signal. (*d*) The co-expression of the proteins in the split YFP experiments in the salivary glands was verified also by western blot. WB, western blot. (*e*) Quantification of (*a*). Data represent mean ± s.d of three independent experiments (*n* = 3), derived from the analysis of approx. 300 cells per sample (3× approx. 300 in total per condition). Shapiro–Wilk’s test was used to test for normality of data distribution. Samples were compared pairwise by Student’s *t*‐test. *p*-values: **p < 0.001 (*p* = 0.0076 and *p* = 0.0085 for Med15 and Med6, respectively).

Based on the results of our previous *in vitro* binding experiments, we tested the Med15, Med17 and Med6 subunits in this assay. The correct expression and subcellular localization of the proteins were confirmed by immunostaining (electronic supplementary material, figure S2). Med17-CYFP showed a mislocalization in the cytoplasm and was therefore excluded from further experiments. When Moesin-NYFP was co-expressed with Med15-CYFP or Med6-CYFP in S2R+ cells, we found a strong YFP signal exclusively in the nucleus (figure 4*a*; quantification in figure 4*e*). The Med19 subunit was introduced as a control Mediator protein. It showed no YFP signal, indicating that Moesin binding to the Med15 and Med6 subunits is specific. The identified subunits represent the head (Med6) and tail (Med15) domains of Mediator, suggesting that Moesin may directly interact with multiple surfaces of the complex and therefore participate in several activities performed by Mediator. Therefore, we selected the Med15 subunit for further functional analysis as it showed interaction in all *in vivo* and *in vitro* assays.

Next, we created transgenic *Drosophila melanogaster* lines in which the Moe-NYFP and Med15-CYFP proteins can be expressed in a tissue-specific manner with the help of the UAS-Gal4 system [31]. Immunostaining experiments revealed the expression and proper subcellular localization of the proteins in salivary glands when the transgenes were driven by the salivary gland specific 3 (Sgs3) Gal4 driver (electronic supplementary material, figure S3). The co-expression of Moe-NYFP and Med15-CYFP proteins in *Drosophila* larval salivary glands or in adult ovaries produced a strong nucleus-specific YFP signal similar to that observed in S2R+ cells (figure 4*b,c*). The giant nuclei of the salivary glands enabled us to determine the subnuclear localization pattern of the interaction between Moe and Med15. We detected clear but diffuse fluorescence in the nucleoplasm, banded pattern on the chromosomes, and also a punctuated pattern within the nucleolus (see the magnification in figure 4*b*). As a control, the co-expression in the salivary glands was verified by western blotting using Myc- and HA-specific antibodies (figure 4*d*). Based on these results, we concluded that through the Med15 tail subunit Moesin is part of the Mediator complex in the nucleus.

### 2.6. Moesin co-localizes with Med15 on activated heat shock genes

**Figure 5.**
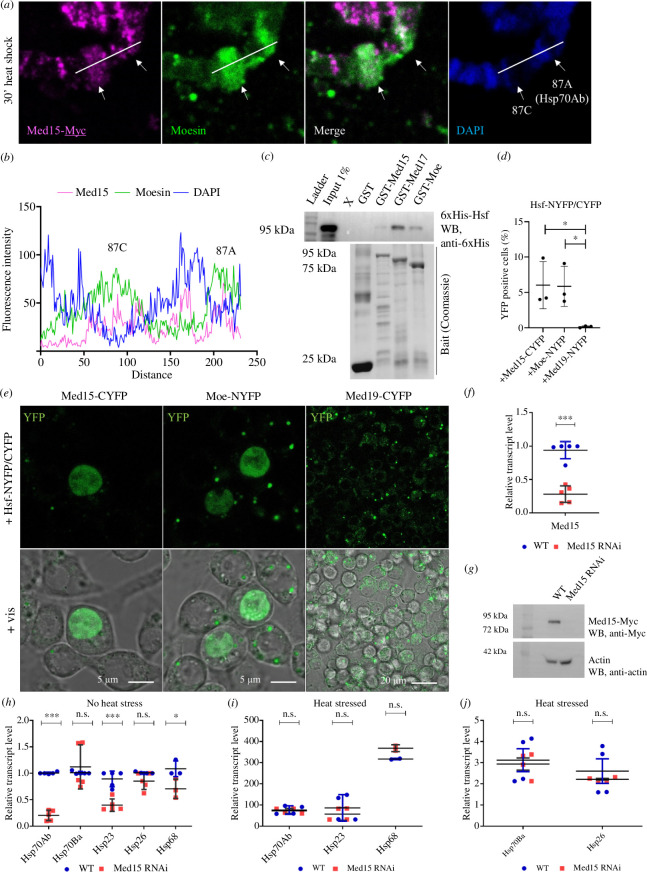
Med15 binds Hsf and is involved in *Hsp* gene expression in *Drosophila.* (*a*) Upon heat shock, Moesin and Med15-Myc proteins accumulate in the heat shock puffs at the 87AC region, which is the cytological location of *Hsp70* genes. (*b*) Quantification of the intensity values of Moesin, Med15 and DAPI stainings in (*a*)*.* (*c*) 6xHis-Hsf (prey) precipitates with GST-Med15 and GST-FERM-Moe (baits) *in vitro*. GST-Med17 was used as a positive control and GST alone as a negative control. WB, western blot. (*d*) Representative images of the split YFP experiments demonstrating the direct interaction of Hsf with Med15 and Moesin proteins in the nucleus of S2R+ cells *in vivo*. Med19 was used as a negative control. (*e*) Quantification of (*d*). Data represent mean ± s.d. of three independent experiments (*n* = 3), derived from the analysis of approx. 300 cells per sample (3× approx. 300 in total per condition). Shapiro–Wilk’s test was used to test for normality of data distribution. Samples were compared pairwise by Student’s *t*‐test. *p*-values: *p < 0.05 (*p* = 0.0375 and *p* = 0.0253 for Med15 and Moe, respectively). (*f,g*) Validation of the *Drosophila* Med15 RNAi strain. The levels of the endogenous Med15 transcript and Med15-Myc protein expressed under the control of the *nanos*>Gal4 driver are significantly reduced upon RNAi silencing. WT, wild-type. (*h*) Silencing of Med15 by RNA interference significantly reduces the expression levels of *Hsp70Ab*, *Hsp23* and *Hsp68* genes (*p* < 0.0001; *p* = 0.0004; *p* = 0.0496, respectively) in the ovaries of unstressed females 2–5 days old. WT, wild-type. (*i,j*) Upon heat stress, there is no significant difference in the expression level of *Hsp* genes in the ovaries of unstressed females 2–5 days old. According to the wide scale of expression levels, we split the results into two graphs. WT, wild-type. (*f,h,i,j*) Graph shows the results from three biological replicates (*n* = 3) with two technical repetitions in each, derived from the analysis of five ovaries per sample (3 × 5 in total per condition). Shapiro–Wilk’s test was used to test for normality of data distribution. Samples were compared pairwise by Student’s *t*‐test. *p*-values: ***p < 0.0001, **p < 0.001, *p < 0.05 and n.s. (not significant): *p* > 0.05.

The Mediator complex has been implicated to play a role in the transcriptional response induced by stress conditions. Certain subunits of the complex (Med15 in *Saccharomyces cerevisiae*, Med17, Med21, Med25 and Med31 in *D. melanogaster*, Med12 in mammalian cells) were already described to be involved in the transcriptional regulation of heat shock protein (*Hsp*) genes through the direct binding of the heat shock factor (Hsf) [21,32,33]. Several lines of evidence support that Moesin is also coupled to the heat shock response. It accumulates in the nucleus upon heat shock and localizes to the sites of actively transcribed *Hsp* genes (called heat shock puffs of the *Drosophila* polytene chromosomes). Furthermore, in chromatin immunoprecipitation (ChIP) experiments, we found that Moesin is present on the promoter and coding region of the *Hsp70* gene [8]. In addition, when Moesin is excluded from the nucleus, the transcript level of several *Hsp* genes significantly decreases [9]. Because the molecular mechanism behind these results was not known, we wanted to test whether Moesin and Mediator may function together in the transcriptional regulation of heat shock genes. First, we carried out immunostaining experiments on larval polytene chromosomes from heat shocked animals, to visualize if Moesin and Mediator co-localize at the heat shock puffs. In these experiments, we stained for the Med15 subunit to detect the complex. After heat shock, both proteins accumulated at the 87AC heat shock puff, which is the cytological location harbouring the *Hsp70* genes (figure 5*a*). The fluorescence intensity measurements of the proteins along the chromosomes confirmed the co-localization (figure 5*b*). This indicates that Moesin may also be present in the Mediator complex that is built up on the actively transcribing *Hsp70* genes.

### 2.7. Med15 is required for proper *Hsp* gene expression in *Drosophila*

Considering the physical interaction between Moesin and Med15 proteins and their co-localization in the heat shock puffs, we investigated their possible role in the transcriptional regulation of *Hsp70* genes. The yeast heat shock factor 1 (Hsf1) binds directly to the heat shock element (HSE) regions located in the promoter of *Hsp* genes [34,35]. It has also been confirmed that Hsf1 facilitates the recruitment of the Mediator complex to the HSE regions by directly binding the Med15 subunit of the tail domain. Accordingly, depletion of yeast Med15 reduces *Hsp* gene expression [21,22].

Because the *Drosophila* Mediator complex has already been shown to be directly involved in the regulation of *Hsp70* gene expression [36], we decided to test whether *Drosophila* Med15, like yeast Med15, is involved in the regulation of Hsf-linked heat shock gene activity. Therefore, we first tested the direct interaction between Med15, Moesin and the *Drosophila* Hsf1 homologue, Hsf, in an *in vitro* pull-down experiment using purified recombinant proteins. We found that 6xHis-Hsf showed direct binding to GST-Med15 and also to GST-Med17, which was used as a positive control in the experiment [32,37], demonstrating that, similarly to the situation in *Saccharomyces*, *Drosophila* Med15 and Hsf physically interact (figure 5*c*). GST-Moesin also showed a clear interaction with Hsf, further confirming a direct role of Moesin in the Hsf pathway (figure 5*c*). To verify the results of the pull-down experiment, we applied the *in vivo* BiFC method in S2R+ cells. In these experiments, the Med19 subunit was used as a negative control, and the expression and correct subcellular localization of the tested proteins were confirmed with immunostaining (electronic supplementary material, figure S4). We observed a nuclear YFP signal with Hsf-NYFP/CYFP, Moe-NYFP and Med15-CYFP, which not only corroborated that Hsf binds Med15 and Moesin but also revealed that Med15 and Moesin are components of the *Hsp* gene-regulating form of Mediator (figure 5*d*, quantification in figure 5*e*). These results suggest that Moe–Med15–Hsf together constitute a complex within a form of Mediator that is involved in the expression of *Hsp* genes.

After confirming the presence of Med15 in Mediator specific for heat shock genes, we tested whether Med15 is really required for *Hsp* expression. To this end, we first validated the *Drosophila* Med15 RNAi line by performing qPCR on total RNA purified from adult ovaries. The qPCR showed that the RNAi induced with the germline-specific *nanos*>Gal4 driver decreased the transcript levels of endogenous Med15 by 70% compared to the wild-type (WT) in unstressed conditions (figure 5*f*). Therefore, we used this driver in the subsequent experiments. We also confirmed by western blotting that the knock down of Med15 caused almost complete depletion of the Med15-Myc protein produced from a transgene (figure 5*g*). We then measured the activity of *Hsp* genes in Med15-silenced ovaries and found that, with the exception of the *Hsp70Ba* and *Hsp26* genes, the expression of the examined *Hsp* genes was significantly reduced, however to varying degrees compared to that of the WT (figure 5*h*). The result was consistent with what we had previously found for Moesin [9] and indicates that *Drosophila* Med15 is required for *Hsp* gene expression and that this is an evolutionarily conserved function of Med15. Surprisingly, when heat stress was applied, Med15 silencing did not significantly reduce *Hsp* gene expression, suggesting that Med15 is involved in maintaining the basic activity of *Hsp* gene expression rather than in increasing it in response to heat shock (figure 5*i,j*).

### 2.8. The association of Moesin with the regulatory region of the *Hsp70Ab* gene depends on Med15

**Figure 6.**
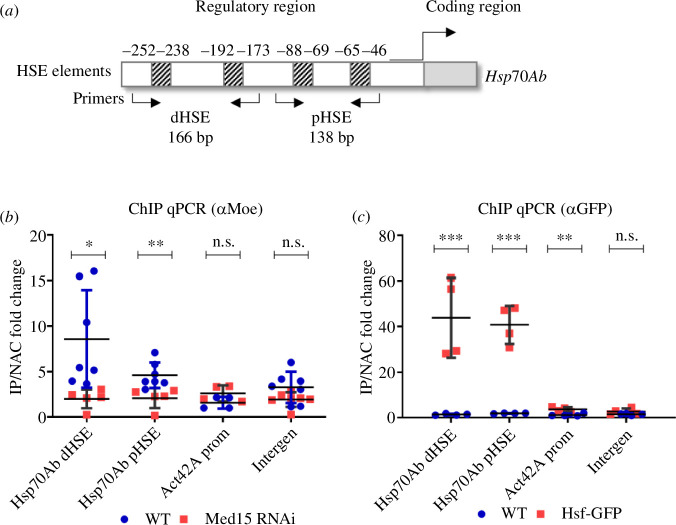
The presence of Moesin in the regulatory region of the *Hsp70Ab* gene depends on Med15. (*a*) Schematic representation of the regulatory region of the *Hsp70Ab* gene. Arrows mark the binding sites of the PCR primers used in the qPCR reactions. (*b*) Result of ChIP qPCR experiments performed with anti-Moesin antibody on the ovaries of wild-type and Med15-depleted, unstressed females 2–5 days old. The level of Moesin on the distal and proximal HSE regulatory regions (dHSE and pHSE, respectively) of the *Hsp70Ab* gene significantly decreases upon Med15 inhibition. On the promoter of the *Act42A* gene or on intergenic region no significant difference was detected. Data represent mean ± s.d. of three independent experiments (*n* = 3) with two technical repetitions in each, derived from the analysis of 125 ovaries per sample (3 × 125 in total per condition). Shapiro–Wilk’s test was used to test for normality of data distribution. Samples were compared pairwise by Student’s *t*‐test. *p*-values: **p < 0.001, *p < 0.05 and n.s. (not significant): *p* > 0.05 (*p* = 0.0234 and *p* = 0.0075 for dHSE and pHSE, respectively). WT, wild-type. (*c*) Control ChIP experiments on wild-type and Hsf-GFP expressing ovaries of female animals 2–5 days old with anti-GFP antibody. The strong accumulation of Hsf-GFP on the regulatory HSE elements of *Hsp70Ab* gene, but not on the promoter of *Act42A* or on the intergenic regions validates the method for the ChIP qPCR assay on ovaries. Data represent mean ± s.d. of three independent experiments (*n* = 3) with two technical repetitions in each, derived from the analysis of 125 ovaries per sample (3 × 125 in total per condition). Shapiro–Wilk’s test was used to test for normality of data distribution. Samples were compared pairwise by Student’s *t*‐test. *p*-values: ***p < 0.0001, **p < 0.001 and n.s. (not significant): *p* > 0.05 (*p* = 0.0028; *p* < 0.0001 and *p* = 0.0053 for dHSE, pHSE and Act42A, respectively). WT, wild-type.

Based on our results and our earlier ChIP data [8], we hypothesized that Moesin localizes to the promoters of the *Hsp* genes in a Med15-dependent manner. To test this idea, we performed ChIP assays coupled with qPCR on ovaries of adult female animals expressing Med15 RNAi under the control of the germline-specific *nanos* promoter. Cross-linked chromatin samples were isolated from Med15-depleted and WT ovaries, and the ChIP experiments were performed using an anti-Moesin antibody. In the qPCR experiments, we tested the presence of Moesin on the regulatory regions of the *Hsp70Ab* gene because its transcript levels significantly decreased when the amount of Moesin was reduced in the nucleus [9] or when Med15 was depleted (figure 5*h*). The examined HSE elements in the regulatory regions of the *Hsp70Ab* gene were defined based on the categories described in [38]. The essence of this is that since the promoter region contains multiple HSE elements, the region was divided into distal and proximal HSE elements (dHSE and pHSE, respectively) relative to the coding region (figure 6*a*). As a control, we measured the level of Moesin on the promoter of the *Act42A* gene, as well as on an euchromatic but transcriptionally inactive (intergenic) region. As an additional control, we also followed the recruitment of Hsf-GFP to the examined regulatory and control regions (figure 6*c*). As Med15 proved to be functional in *Hsp* gene expression in unstressed cells and the Moe–Med15, Moe–Hsf and Med15–Hsf interactions were also detectable in the *in vivo* experiments without heat shock, we conducted the ChIP experiments under unstressed conditions. In addition, many *Hsp* genes are expressed in the *Drosophila* ovary under normal conditions to maintain protein homeostasis [39]. We found that the amount of the Moesin protein significantly decreased in the HSE regions examined when Med15 was depleted, which confirms that the presence of Moesin in the regulatory regions depends on Med15 and that their *in vivo* interaction most likely plays a role in the regulation of *Hsp* genes (figure 6*b*).

### 2.9. Med15 and Hsf interact with actin

**Figure 7.**
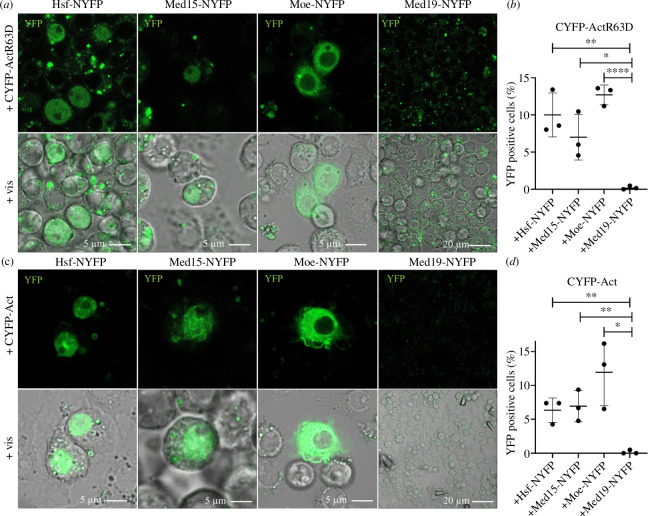
Med15 and Hsf bind actin. (*a*) Split YFP assay showing that the non-polymerizable, R63D point mutant form of Actin5C directly binds Hsf and Med15 proteins specifically in the nucleus of S2R+ cells. Moesin exhibits a predominantly cytoplasmic interaction with actin as both proteins are primarily cytoplasmic and only a small portion of them is present in the nucleus. Med19 was used as a negative control. (*b*) Quantification of (*a*). Data represent mean ± s.d. of three independent experiments (*n* = 3), derived from the analysis of approx. 300 cells per sample (3× approx. 300 in total per condition). Shapiro–Wilk’s test was used to test for normality of data distribution. Samples were compared pairwise by Student’s *t*‐test. *p*-values: ***p < 0.0001, **p < 0.001 and *p < 0.05 (*p* = 0.0046; *p* = 0.0188 and *p* < 0.0001 for Hsf, Med15 and Moe, respectively). (*c*) Representative images of the split YFP assay which shows the interaction of the polymerizable form of actin with Med15 and Hsf in the nucleus of S2R+ cells. Moesin was used as a positive and Med19 as a negative control. (*d*) Quantification of (*c*). Data represent mean ± s.d. of three independent experiments (*n* = 3), derived from the analysis of approx. 300 cells per sample (3× approx. 300 in total per condition). Shapiro–Wilk’s test was used to test for normality of data distribution. Samples were compared pairwise by Student’s *t*‐test. *p*-values: **p < 0.001 and *p < 0.05 (*p* = 0.0043; *p* = 0.0073 and *p* = 0.0146 for Hsf, Med15 and Moe, respectively).

Because Moesin is an actin-binding protein, next we tested whether the Moe–Med15–Hsf complex contains actin. As actin is present in the nuclear complexes primarily in monomeric form [40–42], in the experiments, we applied the non-polymerizable, point-mutant form of actin (Act-R63D). NYFP-tagged Moe, Hsf or Med15 proteins were co-expressed in S2R+ cells along with Act-R63D equipped with an N-terminal CYFP tag, and the Med19 subunit was used as a negative control. We confirmed with immunostaining that the production and intracellular localization of the proteins is normal (electronic supplementary material, figure S5). In the case of Hsf and Med15, we observed clear fluorescent signal in the nuclei of unstressed cells, indicating that G-actin is also present in the Hsf- and Med15-containing complex (figure 7*a*; quantification in figure 7*b*). Since Moesin also showed a YFP signal, this indicated that it is also able to bind the monomeric form of actin, which was already suggested earlier in the case of Ezrin [15]. In contrast to the Hsf and Med15 proteins, in this case, the signal was mainly cytoplasmic and only weakly nuclear because both actin and Moesin are primarily cytoskeletal proteins and only a small portion of them is present in the nucleus.

The existence of actin in the Mediator complex has not been reported before; however, some literature data suggest that polymerized actin binds to the Mediator complex in *S. cerevisiae* and *Xenopus laevis* [43,44]. In addition, Moesin, as a member of the ERM family, is primarily known as an F-actin-binding protein. Therefore, we repeated the split YFP experiments also with the WT, polymerizable form of actin and used Moesin as a control for F-actin binding and Med19 as a negative control. The production and intracellular localization of the proteins was normal (electronic supplementary material, figure S6). Both Med15 and Hsf showed the same nuclear YFP signal as with Act-R63D, which raises the possibility that oligo- or polymeric forms of actin might be present in the Moe–Med15–Hsf complex (figure 7*c*; quantification in figure 7*d*). Moreover, in the case of Med15, we were able to detect a fluorescence pattern reminiscent of an F-actin network in the nucleus, indicating that the Mediator complex may be associated with F-actin filaments.

### 2.10. The binding of Moesin to the Mediator complex is evolutionary conserved

**Figure 8.**
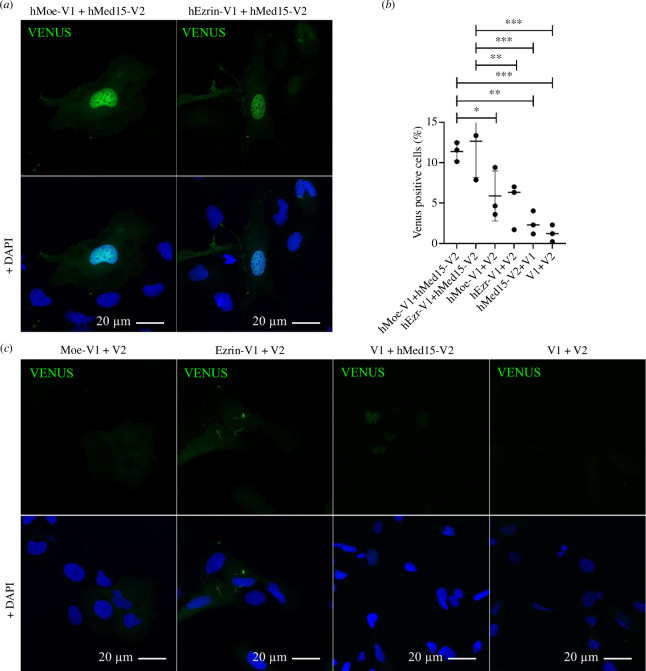
Moesin binding to Mediator complex is evolutionary conserved. (*a*) In split Venus binding assay the human Moesin and Ezrin proteins directly interact with the human Med15 protein in the nuclei of U2OS cells. (*b*) V1 or V2 fragments alone were co-expressed with hMoesin-V1, hEzrin-V1 and hMed15-V2 as negative controls. For an absolute negative control, paired empty vectors were used (V1 + V2). (*c*) Quantification of (*a*) and (*b*). Data represent mean ± s.d. of three independent experiments (*n* = 3). In each experiment, at least 1000 cells were examined. Shapiro–Wilk’s test was used to test for normality of data distribution. Samples were compared pairwise by Student’s *t*‐test. *p*-values: ***p < 0.0001, **p < 0.001, *p < 0.05 and n.s. (not significant): *p* > 0.05.

Since the Med15-Hsf interaction also exists in yeast and all members of the Moe–Med15–Hsf complex are evolutionarily highly conserved, we investigated whether the interaction between Moesin and the Mediator complex is specific to *Drosophila* or is evolutionarily conserved and exists in other species. For this, we subcloned the human ERM proteins hEzrin and hMoesin and hMed15 into human expression vectors tagging with Venus fragments V1 and V2, respectively, to perform a split Venus assay. U2OS cells were co-transfected with pairs of plasmid constructs Moe-V1/Med15-V2 and Ezrin-V1/Med15-V2. As controls, all the three genes of interest were paired with an empty control vector (V1-GW/V2-GW) and as an absolute negative control, paired empty vectors were used. We could observe the same, strong nucleus-specific Venus signal with both ERM proteins, indicating that the binding of Moesin to the Mediator complex through the Med15 subunit is evolutionarily conserved (figure 8*a,b*; quantification in figure 8*c*). The result also reveals that this function can be extended to the ERM protein family, as hEzrin showed the same interaction as hMoesin (figure 8*a*).

## 3. Discussion

**Figure 9.**
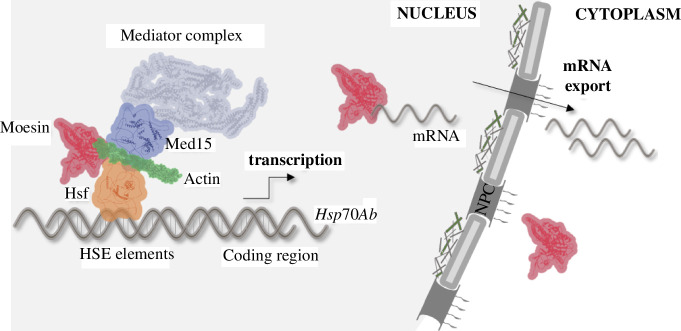
Model depicting the role of Moesin in *Hsp* gene expression. Based on our results, we propose a model according to which Hsf associates with the HSE regions of the *Hsp70Ab* gene promoter, and this targets the Med15 subunit of the tail domain of the Mediator complex to the promoter. Moesin binds directly to Med15 and Hsf, and Moe–Med15–Hsf form a complex which probably binds both the monomeric and the polymerized forms of actin. As ERM proteins are primarily cross-linking proteins, we suggest that the molecular function of Moesin in the Mediator complex might be the stabilization of the complex where it anchors proteins with the help of actin.

Based on our results, we propose a model according to which Hsf associates with the HSE regions of the *Hsp70Ab* gene promoter, and this targets the Med15 subunit of the tail domain of the Mediator complex to the promoter. We found that Moesin binds directly to Med15 and Hsf in unstressed cells, and we suggest that Moe–Med15–Hsf together form a complex which binds both the monomeric and polymeric forms of actin. Since ERMs are primarily cross-linking proteins, we assume that the molecular function of Moesin in Mediator may be to stabilize the complex as it holds proteins together with the help of actin. This function of Moesin is necessary so that the Mediator complex can carry out its activity required for the proper expression of *Hsp* genes. At the same time, the previously described function of Moesin in mRNA export [8] may also contribute to the correct expression of *Hsp* genes (figure 9). It has been reported that yeast Med15 can be recruited to *Hsp* genes independently of the core Mediator complex; therefore, the composition of the rest of the Mediator complex containing Moesin, Med15 and Hsf is an open question today.

The expression of *Hsp* genes under non-stress conditions is essential for the cell since Hsps are pivotal for the proper folding and unfolding of proteins, the assembly of multiprotein complexes, the transport and sorting of proteins to appropriate subcellular compartments and the control of the cell cycle and signalling [45]. The significance of Hsps in a normal physiological state is also supported by the fact that Hsf depletion is lethal in *Drosophila* even in the absence of stress [46]. Therefore, it is tempting to speculate that the Moe–Med15–Hsf complex contributes to ageing and lifespan determination through the regulation of Hsp protein expression under normal physiological condition. At the same time, however, we cannot rule out the possibility that Moesin and Med15 may also be involved in the regulation of the *Hsp* genes in the event of heat stress. The role of Med15 in stress response has already been demonstrated [47], and it is evident also in the case of Moesin, since the transcript levels of *Hsp* genes can only increase to a lesser extent in response to heat shock when the amount of nuclear Moesin is reduced [9]. The idea is also supported by the observation described here that Moesin and Med15 accumulate at the heat shock puffs of the polytene chromosomes (figure 5*a*). It is possible that, upon heat shock, additional proteins are recruited to the complex and they make the complex more stable to ensure robust transcriptional activity, and in this case, Med15 silencing has a less significant effect.

We hypothesize that the Moe–Med15–Hsf interaction may also occur on non-heat shock genes and may play a role in their regulation because [1] the pattern of their physical interaction revealed by the split YFP experiments is not restricted to *Hsp* loci and [2] Moe and Med15 co-localized along the entire chromosomes, not only in regions containing heat shock genes. Interestingly, *Drosophila* Hsf is also required for developmental processes mediated by non-*Hsp* genes, such as oogenesis and early larval development [46]. Consistent with this, *Drosophila* Hsf has been shown to be present not only on promoter regions but also on exons and introns, as well as on genes not regulated by heat stress. In *Drosophila*, 434 genes contain Hsf binding sites, and interestingly, a substantial number of these genes do not respond to heat stress [48].

One type of genes that do not respond to heat shock, but require Hsf binding, thus the Moe–Med15 interaction, may be genes that are regulated by hormone induction. We previously demonstrated that Moesin accumulates in the nucleus upon growth hormone (ecdysone) treatment [8], and the direct interaction of Mediator complex members (Med1, Med14, Med15, Med26 and Med27) and the ecdysone receptor in the nucleus has already been demonstrated in *Drosophila* [49,50]. In addition, Moesin and members of the Mediator complex also co-localized in the ecdysone puffs of the polytene chromosomes in our experiments (not shown here). These findings support the idea that the function of the Moe–Med15–Hsf interaction is not limited to *Hsp* genes and may also be required for the transcription of developmental genes. Future experiments would be required to clarify the exact role of the Moe–Med15–Hsf interaction in the regulation of non-heat shock genes.

Since both Moesin and Mediator perform very versatile activities in the cell, it is easy to imagine that their joint tasks are not restricted to heat shock or hormone-induced gene transcription, but together they are actively involved in other levels of gene regulation. This is indicated by our observation that significant overlapping extrachromosomal staining is observed between Moesin and Mediator subunits (figure 3*a*, marked with white stars), suggesting a possible role in post-transcriptional processes, such as mRNA editing and export, as previously described for both Moesin [8] and the Mediator complex [51,52]. In addition, our results also suggest that Moesin can bind Mediator subunits other than Med15, such as the Med6 and Med17 components of the head domain (figures 2 and 4*a*). Since Med6 and Med17 are involved in different functions of the Mediator complex than Med15, their interaction with Moesin may be required for other functions of the complex beyond heat shock- or hormone-induced gene transcription. Furthermore, Moesin and Med15 also showed an obvious interaction in the nucleolus of salivary glands in split YFP experiments (figure 4*b*), further expanding the possible multiple functions of Moesin with the Mediator complex.

The consensus HSE sequence is remarkably conserved from *Saccharomyces* through *Drosophila* to human [34,53,54]. As in our experiments, we found that, like yeast Hsf1, *Drosophila* Hsf also binds Med15, it seems that not only the HSE elements themselves but also this interaction on the HSE element is evolutionary conserved. However, our results show that the binding of Moesin, Med15 and Hsf also occurs in non-stressed cells, which interestingly contradicts the model established in *Saccharomyces*, according to which Mediator regulates *Hsp* expression only in acute heat stress, but not in chronic heat stress or under normal conditions [21]. In *Saccharomyces*, actin function is excluded from Hsf1-mediated *Hsp* gene activity, as described by Rubio *et al.* [55]. Since there are no ERM proteins in yeast, we believe that the role of actin in the regulation of *Hsp* gene activity could have appeared together with the ERM proteins in metazoan cells [6]. Consequently, the Mediator complex may have achieved its novel function in maintaining the basal levels of *Hsp* genes, which is realized through the cooperation with ERMs and actin, in multicellular animals with differentiated tissues.

The human orthologue of Med15 is coupled with pathological conditions, such as cancer [56–59] lipid metabolism disorders [60,61], autism [62], schizophrenia [63] or DiGeorge syndrome, which is one of the most frequent genetic deletions coupled with intellectual disability [64–67]. The N-terminal disordered poly Q-rich domain of Med15 was shown to organize itself into prion-like *β*-sheet amyloid structures, which may have a pathological role, but it is also known that this structure can play an epigenetic transcriptional regulatory role in the cell [68,69]. Therefore, it is essential to understand the exact molecular mechanisms and roles behind the Moesin–Med15 nuclear interaction in the future.

In addition, there is also a disordered region at the C-terminus of *Drosophila* Moesin [70], which makes it even more likely that Moesin is part of the molecular condensate formed by the well-known phase-separating ability of the Mediator complex [71] and thereby may contribute to the pathological conditions linked to the Mediator complex.

## 4. Material and methods

### 4.1. Cell maintenance, transfection and immunostaining

The S2R+*Drosophila* cell line (DGRC Stock 150; RRID:CVCL_Z831) was maintained in Schneider’s *Drosophila* medium (Biowest, L0207-500) supplemented with 10% fetal bovine serum (Biowest, Fetal Bovine Serum French Origin, S1820-500 Lot.: S15960S1820) and 1% antibiotics (Capricorn, Pen/Strep 100 ×, PS-B) at 26°C. To transfect the cells, the Effectene Transfection Reagent Kit (Qiagen, 301425) was used according to the manufacturer’s instructions. For immunostaining experiments, 2 × 10^5^ cells/well were used and incubated for 2 days after transfection on a round coverslip of 12 mm diameter (Epredia, CB00120RA120MNZ0) in 24-well plates (Sarstedt, 83.3922). For the split YFP experiments, 8 × 10^5^ cells/well were transfected in 35 mm glass bottom dishes (Thermo Scientific, 150680) and incubated for 2 days after transfection.

For immunostainings of S2R+ cells, the method described in [8] was followed. In brief, cells were fixed in 4% paraformaldehyde-PBS (PFA-PBS) solution, washed three times in 1 × PBS and permeabilized with PBT (PBS, 0.1% Triton X-100; Sigma-Aldrich, X100) at RT. Blocking was carried out in PBT-N solution (PBT, 1% BSA [Sigma-Aldrich, A4503], 5% FCS [Merck, N4637]) for at least 1 h at RT. Cells were incubated with the anti-FLAG M2 (1 : 500, Sigma-Aldrich, F1804; RRID:AB_10707327), anti-GFP (1 : 500, Life Technologies, A6455; RRID:AB_221570), anti-Myc (1 : 500, Santa Cruz Biotechnology, sc-40; RRID:AB_627268) or anti-HA (1 : 500, Sigma-Aldrich, H6908; RRID:AB_260070) primary antibody overnight at 4°C. After washing two times with PBT, samples were incubated with the fluorescently labelled secondary antibody (1 : 600, Invitrogen Molecular Probes) supplemented with DAPI (0.2 µg ml^−1^, Sigma-Aldrich) for 2 h in dark at RT. Cells were mounted in 7 µl of Fluoromount-G medium (Southern Biotech, 00-4958-02) and imaged with a Zeiss LSM 800 confocal microscope using a 63.0 × (1.40 NA) oil objective. Live imaging of split YFP experiments performed in S2R+ cells was carried out with a Zeiss LSM 800 confocal microscope using a 63.0 × (1.40 NA) oil objective.

Split Venus experiments with human proteins were carried out in U2OS human osteosarcoma cell line (Sigma-Aldrich, 92022711; RRID:CVCL_0042). For transfection, Xfect transfection reagent (Takara, 631318) was used following the manufacturer’s instructions; 48 h after transfection, the samples were fixed with 4% paraformaldehyde, stained with DAPI and imaged with a Zeiss LSM 800 confocal microscope using a 63.0 × (1.40 NA) oil objective. The used cell line was tested negatively to Mycoplasma infection.

### 4.2. Evaluation of split YFP assays and statistical analysis

The split YFP experiments were evaluated on living *Drosophila* S2R+ cells using the image analysis software (ZEN 3.4; RRID:SCR_013672) of the Zeiss LSM 800 confocal microscope. Nuclei with a detectable fluorescence signal were considered as YFP positive cells, regardless of the intensity of the signal. Cells were manually counted in six to eight randomly selected microcopy fields of view, each containing approx. 50 cells, in three individual biological repetitions. We considered the 3× approx. 300 cells in total per condition a representative amount of sample for quantification. The position of the nucleus was determined based on the bright-field microscopy photos. The percentage of YFP positive cells was calculated relative to the total cell number in all experiments.

The BiFC experiments with human proteins were performed on fixed U2OS cells. Samples were analysed with an ImageXpress Micro Confocal high-throughput microscope (Molecular Devices) using the MetaXpress software package (Molecular Devices, RRID:SCR_016654). Thirty-six photos were taken from each sample, using a 40 × Plan Fluor ELWD objective. Two filters were used for scanning: DAPI: 377/447 nm; and FITC: 475/536 nm. For all filters, the excitation times were 100 ms. Total cell number was determined by counting DAPI-stained nuclei. The minimum intensity limit above local background was set to 1000 grey level. In each biological repetition, at least 1000 cells were examined (3 × 1000 in total per condition). Localization of the Venus signal was determined with the following method. The Venus signal had to be higher than 2500 grey level, to distinguish the signal from the background. Cells were classified with only nuclear localization, if the correlation coefficient of the Venus and DAPI signals was equal, or above 0.8. Cells were classified with cytoplasmic localization, if the quotient of the cytoplasmic and nuclear (outer and inner) median of the Venus signal was equal or greater than 1.2. The percentage of YFP positive cells was calculated relative to the total cell number in all experiments.

Shapiro–Wilk’s test was used to test for normality of data distribution. Student’s *t*-tests were performed for pairwise comparisons. Graphs showing the mean ± s.d. were created with GraphPad Prism 9.1.2 (GraphPad Software, RRID:SCR_002798). Statistical significance is marked with ***p < 0.0001, **p < 0.001, *p < 0.05 and n.s. (not significant): *p* > 0.05.

### 4.3. Cloning of expression vectors for cell transfection

For C-terminal GFP/HA/Flag tagging the full-length CDS (DGRC Gold collection) of Moesin (SD10366), Med15 (GH03922) and Med6 (LD15729) were PCR amplified using gateway cloning-compatible primer pairs (table 1), and the resulting PCR products were recombined into pDONR221 vector (Thermo Fisher Scientific, 12536017) according to the manufacturer’s instructions (Thermo Fisher Scientific, Gateway BP Clonase II Enzyme Mix, 11789020). All constructs were sequence-verified and subcloned into the pAWG (DGRC Stock 1072; RRID:DGRC_1072), pAWH (DGRC Stock 1096; RRID:DGRC_1096) or pAWF (DGRC Stock 1112; RRID:DGRC_1112) *Drosophila* gateway expression vectors according to the manufacturer’s instructions (Thermo Fisher Scientific, Gateway LR Clonase II Enzyme Mix, 11791100).

**Table 1.** Primers.

name	sequence 5′–3′
Hsp70Ab dHSE Fw	CCTAGCTCTGCGATTATCTCTAAC
Hsp70Ab dHSE Rev	CGAATAGAGAATAACGGCCAGAG
Hsp70Ab pHSE Fw	GTGACTCTCCCTCTTTGTACTATT
Hsp70Ab pHSE Rev	GTCGACGAAGCTCCTCTATTT
Act42A Fw	CGCTCTCCAGTCTTCACCGTTT
Act42A Rev	CCGCTGCACACACTTAGCACAAT
Intergen Fw	ACACTGCGAGCGCCTCACACGC
Intergen Rev	CCTAGGTGAATGTGCGGCACAC
Hsp70Ab gene Fw	AAGGGTGAGTCCAAGAGATTTG
Hsp70Ab gene Rev	TGATGACTGCGTCTGTGATG
Hsp70Ba gene Fw	GATGGTGCTGACCAAGATGAA
Hsp70Ba gene Rev	CGCTGAGAGTCGTTGAAGTAAG
Hsp23 gene Fw	AGCGAACTGGTGGTCAAAG
Hsp23 gene Rev	GGACAAAGTGACGAGTGATGAA
Hsp26 gene Fw	TGGTGGACGACTCCATCTT
Hsp26 gene Rev	TAGCCATCGGGAACCTTGTA
Rp49 Fw	CCGCTTCAAGGGACAGTATCTG
Rp49 Rev	ATCTCGCCGCAGTAAACGC
Moesin cDNA Fw	GGGGACAAGTTTGTACAAAAAAGCAGGCTTCACCATGTCTCCAAAAGCGCTA
Moesin cDNA Rev	GGGGACCACTTTGTACAAGAAAGCTGGGTCGCTGGACATGTTCTCAAAC
FERM-Moe cDNA Rev	GGGGACCACTTTGTACAAGAAAGCTGGGTCGCTATCGCCGTTCGTCAGCTC
Med15 cDNA Fw	GGGGACAAGTTTGTACAAAAAAGCAGGCTTCACCATGACCGAGGACTGG
Med15 cDNA Rev	GGGGACCACTTTGTACAAGAAAGCTGGGTCGCTGGAAACTCCCAGCAAAGT
Med6 cDNA Fw	GGGGACAAGTTTGTACAAAAAAGCAGGCTTCACCATGGCCAGCCGACAG
Med6 cDNA Rev	GGGGACCACTTTGTACAAGAAAGCTGGGTCGCTGGACTTGCTCTTCTTTTC
Med17 cDNA Fw	GGGGACAAGTTTGTACAAAAAAGCAGGCTTCACCATGTCGAATTCCGTG
Med17 cDNA Rev	GGGGACCACTTTGTACAAGAAAGCTGGGTCGCTGGAGGCCGTGTTACTTGT
Med19 cDNA Fw	GGGGACAAGTTTGTACAAAAAAGCAGGCTTCACCATGATGAGCAACTAC
Med19 cDNA Rev	GGGGACCACTTTGTACAAGAAAGCTGGGTCGCTGGAAAACTGCGACATAAG
Hsf cDNA Fw	GGGGACAAGTTTGTACAAAAAAGCAGGCTTCACCATGATGTCCAGGTCGCGTT
Hsf cDNA Rev	GGGGACCACTTTGTACAAGAAAGCTGGGTCGCT GGA CAACTCGTGACGTGGC
ActR63D cDNA Fw	GGGGACAAGTTTGTACAAAAAAGCAGGCTTCACCATGTGTGACGAAGAAGTT
ActR63D cDNA Rev	GGGGACCACTTTGTACAAGAAAGCTGGGTCGCTTTAGAAGCACTTGCGG
FERM-Moe pGEX6P-1 Fw	CCCGGGTCGACTCGAC ATGTCTCCAAAAGCGCTAAA
FERM-Moe pGEX6P-1 Rev	GATGCGGCCGCTCGATTAATCGCCGTTCGTCAGCT
Med15 pGex6P-1 Fw	CCCGGGTCGACTCGACATGACCGAGGACTGGCAGAG
Med15 pGex6P-1 Rev	GATGCGGCCGCTCGACTAAACTCCCAGCAAAGTGG
Med17 pGex6P-1 Fw	CCCGGGTCGACTCGACATGTCGAATTCCGTGAATAT
Med17 pGex6P-1 Rev	GATGCGGCCGCTCGATCAGGCCGTGTTACTTGTCA
hMoe cDNA Fw	GGGGACAAGTTTGTACAAAAAAGCAGGC TTC ACCATGCCCAAAACGATCAGT
hMoe cDNA Rev	GGGGACCACTTTGTACAAGAA AGCTGGGTCGCTGGACATAGACTCAAATTCGTC
hEzrin cDNA Fw	GGGGACAAGTTTGTACAAAAAAGCAGGC TTCACCATGCCGAAACCAATCAAT
hEzrin cDNA Rev	GGGGACCACTTTGTACAAGAAAGCTGG GTCGCTGGACAGGGCCTCGAACTCGTC
hMed15 cDNA Fw	GGGGACAAGTTTGTACAAAAAAGCAGGCTTCACCATGGACGTTTCCGGGCAA
hMed15 cDNA Rev	GGGGACCACTTTGTACAAGAAAGCTGGGTCGCTGGAGGCGGCTGAGAGGCAGGCC

For the split YFP experiments in S2R+ cells, the full-length CDS of Moesin, Med15, Med17 (SD10038), Med6, Med19 (LD41395), Hsf (BS29431) and Actin5C were PCR amplified using a primer pair with overhanging gateway recombination sites (table 1). The resulting PCR products were recombined into pDONR221 entry vector. All constructs were sequence-verified and then subcloned into split YFP tagging gateway vectors [72] in which the inducible UAS promoter was replaced by the promoter of the *Act5C* gene to ensure constitutive protein expression (pAct5C-W-Myc-NYFP, pAct5C-W-HA-CYFP, pAct5C-NYFP-Myc-W and pAct5C-CYFP-HA-W).

For human expression vectors, we PCR amplified the full-length CDS of hMed15 (abm, Med15 ORF vector, 282980110000), and hEzrin and hMoesin (gifts from Dorothy Crouch, University of Dundee, UK) using a primer pair with overhanging gateway recombination sites and recombined them into pDONR221 vector. All constructs were sequence-verified and then subcloned into pcDNA GW-V1 or pcDNA GW-V2 vectors. Primers are listed in table 1.

### 4.4. Protein co-immunoprecipitation assay from S2R+ cells

Cells grown in Petri dishes of 60 mm diameter were trypsinized and collected by centrifugation. The pellet was washed 2X with ice-cold PBS then resuspended in 100 µl Lysis Buffer (20 mM Tris pH 7.5, 200 mM NaCl, 10% glycerol, 0.5 mM EDTA, 0.5% NP-40) containing freshly added proteinase inhibitor cocktail (Protease Inhibitor Cocktail Tablets, Roche) and incubated on ice for 1 h. For lysis control, 5% of the protein samples were saved. Total protein concentration was measured with the Bradford method (Pierce Coomassie Plus Assay Kit, Thermo Fisher 23236) by comparing to BSA protein standards, and equal protein amounts were used in the no antibody control (NAC) and IP samples. NAC and IP samples were diluted with Lysis Buffer and the antibody was added to them. After gentle rotation at 4°C overnight, 20 µl of Protein G magnetic beads (BioRad 161–4021) was added, previously washed with Lysis Buffer. Samples were rotated for 1 h and 15 min at 4°C, and then, the beads were washed with Lysis Buffer for 2 × 10 min. To elute the proteins, the beads were resuspended in 20 µl of 2 × SDS sample buffer and boiled for 5 min and spun for 5 min with 10 000 *× g* at RT.

### 4.5. Blue native polyacrylamide gel electrophoresis

The experiment was carried out according to the method described in [73]. Briefly, S2R+ cells were transfected with Med7-FLAG and Med15-FLAG expressing DNA constructs. Two days after transfection, nuclear protein fraction was isolated from the cells followed by a dialysis in BN-Dialysis buffer (20 mM Bis-Tris, 500 mM aminocaproic acid, 20 mM NaCl, 2 mM EDTA, 10% glycerol, 0.1% Triton-X 100 and 1 × protease inhibitor cocktail) at 4°C overnight. Protein complexes were separated at 4°C on a native 4–10% gradient polyacrylamide gel using cathode (15 mM Bis-Tris, 50 mM Tricine, 0.02% Coomassie blue G250, pH 7.0) and anode buffers (50 mM Bis-Tris pH 7.0). After running, the BN-PAGE gel slice was incubated for 10 min at RT in 2 × SDS sample buffer, then briefly boiled in a microwave oven and incubated hot for another 15 min. For the second-dimension SDS-PAGE, the gel slice treated as described above was placed on the top of a standard 10% SDS gel, and electrophoresis and western blotting were performed according to standard protocols, and then the desired proteins were visualized using antibodies.

### 4.6. Sample preparation for the mass spectrometric identification of interacting proteins

S2R+ cells were transfected in a 10 cm Petri dish with DNA constructs expressing either Moesin-GFP or Moesin-HA. Two days post-transfection, the cytoplasmic and nuclear protein fractions were isolated as described previously [8], with the exception that Buffer C was replaced with 20 mM Tris buffer (20 mM Tris, pH 7.5, 150 mM NaCl, 10% glycerol, 0.5 mM EDTA) containing 1 × protease inhibitor cocktail (Complete EDTA-free Protease inhibitor cocktail tablets, Sigma-Aldrich, 11873580001) and 0.5% NP-40. Total protein extracts (nuclear and cytoplasmic) were immunopurified using anti-GFP (Miltenyi Biotec MACS GFP Isolation Kit, 130-091-125) or anti-HA (Miltenyi Biotec MACS HA Isolation Kit, 130-091-122) antibody-coupled magnetic beads and digested on a column (Miltenyi Biotec, 130-042-201) with trypsin (Thermo Fisher Scientific, Pierce Trypsin Protease MS Grade, 90057).

### 4.7. Mass spectrometry and data interpretation

The tryptic peptide mixture was desalted prior to LC-MS/MS analysis on a C18 ZipTip (Omix C18 100 μl tips, Varian), and the purified peptide mixture was analysed by LC-MS/MS using a nanoflow RP-HPLC (LC program: linear gradient of 3–40% B in 100 min, solvent A: 0.1% formic acid in water, solvent B: 0.1% formic acid in acetonitrile) online coupled to a linear ion trap-Orbitrap (Orbitrap-Fusion Lumos, Thermo Fisher Scientific) mass spectrometer operating in positive ion mode. Data acquisition was carried out in data-dependent fashion, the 20 most abundant, multiply charged ions were selected from each MS survey for MS/MS analysis (MS spectra were acquired in the Orbitrap, and CID spectra in the linear ion trap).

Raw data were converted into peak lists using the in-house Proteome Discoverer (v 1.4) and searched against the UniProt *D. melanogaster* database (downloaded 2019.06.12, 52 524 proteins) including additional proteins identified from the previous Swiss-Prot search (protein score > 50) using our in-cloud Protein Prospector search engine (v. 5.15.1) with the following parameters, enzyme: trypsin with maximum two missed cleavage; mass accuracies: 5 ppm for precursor ions and 0.6 Da for fragment ions (both monoisotopic); fixed modification: carbamidomethylation of Cys residues; variable modifications: acetylation of protein N-termini; Met oxidation; cyclization of N-terminal Gln residues, allowing maximum two variable modifications per peptide. Acceptance criteria: minimum scores 22 and 15; maximum *E* values 0.01 and 0.05 for protein and peptide identifications, respectively. Spectral counting was used to estimate relative abundance of individual proteins in the no-antibody negative control and in the anti-GFP/anti-HA immunopurified samples [74].

The final score for each protein was calculated using the data of six biological replicates (three of Moesin-GFP and three of Moesin-HA replicates) and by multiplying the fold change of peptide count by coverage percentage and IP efficiency. Peptide count is the number of different detected peptides in the mass spectrum that derive from the same protein. Coverage percentage (or sequence coverage %) is the percentage of the protein sequence that is covered by the identified peptides. IP efficiency was calculated by the peptide count enrichment of Moesin compared to the NAC.

### 4.8. Generation and maintenance of *D. melanogaster* transgenic stocks

Fly (RRID:NCBITaxon_7227) strains were maintained and crosses were carried out on standard cornmeal, yeast, sucrose *Drosophila* medium at 25°C. Stocks number 32 517 (y^1^ sc* v^1^ sev^21^; P{TRiP.HMS00522}attP2), 6870 (w[1118]; P{Sgs3-GAL4.PD}TP1), 4441 (y^1^ w*; P{GAL4-nanos.NGT}40) and 66 741 (w^1118^; PBac{Hsf-GFP.FPTB}VK00033) were obtained from the Bloomington *Drosophila* Stock Center. To generate flies expressing full-length Moesin-HA-CYFP, the Moesin coding region was PCR amplified from the CDS using a primer pair with overhanging gateway recombination sites (table 1). The resulting PCR product was recombined into pDONR221 plasmid, sequence-verified then subcloned into a gateway vector equipped with an inducible promoter and providing C-terminal CYFP and HA epitope tagging [72].

For Med15-Myc and Med15-Myc-NYFP expressing flies, the Med15 coding region was PCR amplified from the CDS using a primer pair with overhanging gateway recombination sites (table 1). The resulting PCR product was recombined into pDONR221 plasmid, sequence-verified and subcloned into the pPWM (DGRC Stock 1110; RRID:DGRC_1110) gateway vector (*Drosophila* gateway vector collection) or the gateway vector equipped with an inducible promoter and providing C-terminal NYFP and Myc epitope tagging [72]. Transgenic flies were generated at the *Drosophila* Injection Facility of the BRC Szeged, by using lines carrying attB recombination sites.

### 4.9. Polytene chromosome preparation and statistical analysis

Dissected salivary glands were fixed in 45% acetic acid-PBS for 5 min at RT, then squashed on a Poly-l lysine (Sigma, P8920)-coated slide under a coverslip. The sample was frozen in liquid nitrogen, and the coverslip was immediately removed with a blade. Blocking and immunostaining were carried out as described above. All steps were performed in a humidity chamber. Primary antibodies and the dilutions used were: rabbit anti-Moe 1:1000 [9], mouse anti-Myc 1:200 (sc-40, Santa Cruz Biotechnology) and mouse anti-HA (Sigma-Aldrich H3663; RRID:AB_262051). The antibodies against Med12 and Med13 were kind gifts from the Treisman laboratory (The Francis Crick Institute, UK), and against the Med17, Med26 and Med27 proteins from the Michael Marr laboratory (Brandeis University, USA). Imaging was carried out with a Zeiss LSM 800 confocal microscope using a 63.0 × (1.40 NA) oil objective.

For quantitation, pixel intensity measurements along manually fitted lines were performed with the Fiji software (RRID:SCR_002285), and the graph showing the distribution of the signals was created with GraphPad Prism 9.1.2 (GraphPad Software).

### 4.10. Salivary gland and ovary dissection, live imaging

Dissection of third-stage wandering larvae or adult females was performed in Schneider’s *Drosophila* medium. The dissected larval salivary glands or adult ovaries were carefully transferred into a 35 mm glass bottom dish containing Schneider’s medium and immediately imaged with a Zeiss confocal microscope (LSM 800) using a 63.0 × (1.40 NA) oil objective.

### 4.11. Coupled *in vitro* transcription and translation reaction

The IVTT experiments were carried out according to the previously published protocol [75] using the TnT Quick Coupled Transcription/Translation System (Promega, L1170). Briefly, in 15 μl reaction, volumes 90 ng of template DNA were used in each reaction, and ^35^S-Methionine (Perkin Elmer, NEG709A001MC) was added to the reaction to radioactively label the proteins. For input controls, 0.5 μl from each reaction was mixed with 1 × SDS sample buffer, and the rest were used in the GST-IVTT pull-down experiments; 1 × SDS sample buffer was diluted from 5 × stock (300 mM Tris pH 6.8, 32% glycerol, 10% SDS, 0.5 M DTT, 0.2% bromophenol blue). The following clones obtained from the DGRC Gold Collection were used to express Mediator proteins driven by the T7 promoter (vector names are in parentheses): Med8-FI07210 (pFLC-1), Med11-GH01072 (pOT2), Med15-GH03922 (pOT2), Med16-FI01002 (pFLC-1), Med17-SD10038 (pOT2), Med22-RE16720 pFLC-1), Med23-IP14638 (pOT2), Med27 RE51713 (pFLC-1) and Med31 LD35644 (pOT2). The Med6 cDNA was subcloned from the pBS SK- (LD15729) plasmid into pHY22 vector [76] which contains the T7 promoter. The reaction mixes were incubated at 30°C for 2 h.

### 4.12. Cloning of expression vectors for recombinant protein production

For N-terminal GST tagging, the CDS of Moesin, Med15 and Med17 were PCR amplified and the products were cloned into the pGex6P-1 vector (Cytiva, 28-9546-50) linearized with the XhoI enzyme using the In Fusion HD Cloning Kit (Takara, 639649) according to the manufacturer’s instructions. For C-terminal 6xHis, tagging the CDS of Moe and Hsf was PCR amplified with a primer pair containing overhanging gateway recombination sites (table 1) and the product was recombined into pDONR221 plasmid then subcloned into the pDEST17 gateway vector (Thermo Fisher Scientific, 11803012). In the case of Moesin, we applied primers amplifying the coding region corresponds to amino acids 1–484 (FERM-Moe, respectively), to avoid the closed and inactive structure of Moesin in the binding experiments. All constructs were sequence verified before used for protein expression.

### 4.13. Purification of recombinant GST- and His-tagged proteins from bacteria

pGex6P-1 (GST alone) or the FERM-Moe/Med15/Med17-pGex6P-1 constructs were transformed into *E. coli* SixPack [77] competent cells. The starter culture was grown overnight in 3 ml of LB media supplemented with 100 μg ml^−1^ Carbenicillin at 37°C, and on the next day in a 1:200 dilution, it was further grown in a volume of 50 ml medium until the culture reached OD = 0.6–0.8. The protein expression was induced with 0.5 mM IPTG (Sigma-Aldrich, I6758-1G) overnight at 18°C. The next day, the bacteria were collected with centrifugation (7700 × *g*, 5 min, 4°C), and the pellet was washed with Lysis Buffer (50 mM Tris-HCl pH 7.4, 5 mM DTT, 50 mM NaCl, 5 mM EDTA, 10% glycerol and protease inhibitor cocktail). All subsequent steps were carried out at 4°C. After the washing step, the pellet was resuspended in 10 ml of Lysis Buffer and sonicated on ice for 2 × 4 min by using an ultrasonic homogenizer (Biologics, Model 3000). The lysate was incubated on ice for 30 min with 10% Triton X-100. After centrifugation for 10 min at 7700 × *g*, the supernatant was incubated for 3 h with 250 µl of affinity beads (Glutathione Sepharose 4B, Cytiva, 17-0756-01) in a chromatography column (Bio-Rad, 731–1550). Then the beads were washed with 5 ml of Wash Buffer1 (50 mM Tris-HCl pH 7.4, 5 mM DTT, 400 mM NaCl, 10% glycerol) and 2 × 5 ml of Wash Buffer2 (50 mM Tris-HCl pH 7.4, 5 mM DTT, 50 mM NaCl, 5 mM MgCl_2_, 10 mM KCl, 5% glycerol). The proteins immobilized onto the affinity beads were stored in Storage Buffer (50 mM Tris-HCl pH 7.4, 150 mM NaCl, 1 mM DTT, 60% glycerol) at −20°C before use in pull-down assay. For protein elution, beads were incubated with 1.1 ml of Elution Buffer (Wash Buffer2 supplemented with 25 mM reduced glutathione (Sigma-Aldrich, G4251, pH 8.0)) for 30 min on a rocker-shaker. The buffer was changed to Storage Buffer2 (50 mM Tris-HCl pH 7.4, 150 mM NaCl, 1 mM DTT, 5% glycerol), and the samples were concentrated to the volume of approx. 250 µl by centrifugation for 35 min at 7700 × *g* with a centrifugal concentrator (Amicon Ultra filter units 4K: Millipore, UFC800324). The concentrated protein preparations were divided into aliquots and stored at −80°C.

For the purification of 6xHis-tagged proteins, the same method was followed except that HisPur Cobalt Resin (Thermo Scientific, 89964) or Ni-NTA (Thermo, 88221) beads, and Lysis Buffer (100 mM NaH_2_PO_4_ pH 8.0, 150 mM NaCl, 5 mM Imidazole (Sigma-Aldrich, I5513)), Wash Buffer (100 mM NaH_2_PO_4_ pH 8.0, 300 mM NaCl, 20 mM Imidazole) and Elution Buffer (100 mM NaH_2_PO_4_ pH 8.0, 300 mM NaCl, 300 mM Imidazole) were used.

### 4.14. GST-IVTT and GST-6xHis pull-down assay

GST-IVTT pull-down was performed as described previously [75]. In brief, immobilized GST-tagged proteins were washed with Wash Buffer1 (50 mM Hepes pH 7.5, 150 mM NaCl, 2 mM MgCl_2_, 1 mM EGTA, 1 mM DTT, 0.1% Triton X-100) and resuspended in Wash Buffer+ (Wash Buffer1 supplemented with 1 × protease inhibitor cocktail and 0.5 mg ml^−1^ BSA). Equal amount of immobilized bait proteins was used, which we estimated by SDS-PAGE followed by Coomassie brilliant blue staining. Next, the ^35^S-methionine-labelled proteins synthesized by IVTT or the purified His-tagged recombinant proteins (prey) were mixed with the immobilized GST-tagged baits and incubated for 1.5 h at 4°C with rotation. Then, the beads were washed three times with Wash Buffer1 and three times in Wash Buffer2 (Wash Buffer1 supplemented with 50 mM NaCl and 0.1% Triton X-100) for 5–5 min each at 4°C with rotation. Finally, the beads were collected in 1 × SDS sample buffer and boiled for 5 min.

### 4.15. Western blot and autoradiography

Protein samples were fractionated by 10% SDS-PAGE, and proteins were transferred to PVDF membrane (Millipore Transfer Membranes Immobilon-P, IPVH00010, PVDF 0.45 µm) using 300 mA for 1 h at 4°C. Blocking was performed in 5% milk powder-TBST (25 mM Tris pH 7.5, 150 mM NaCl, 0.1% Tween-20) for 2 h at RT, and then the membrane was incubated with the primary antibodies anti-Moe (rabbit, 1 : 10.000 [9]), anti-GFP (rabbit, 1 : 5000, Life Technologies A6455), anti-FLAG M2 (mouse, 1 : 1000, Sigma-Aldrich F1804), anti-HA (mouse, 1 : 1000, Sigma-Aldrich H3663), anti-Myc (mouse, 1 : 1000, Santa Cruz Biotechnology 9E10), anti-His-HRP (1 : 10 000, Sigma-Aldrich A7058-1VL, Monoclonal Anti-polyHistidine-Peroxidase antibody produced in mouse; RRID:AB_258326) and anti GST-HRP (1 : 10 000, Millipore 16–209; RRID:AB_310805) for 1 h at RT or overnight at 4°C. The membranes were washed two times in TBST for 20 min and incubated with the HRP-conjugated secondary antibody [Jackson ImmunoResearch, 111-035-144, RRID:AB_2307391 (anti-rabbit); 115-035-003, RRID:AB_10015289 (anti-mouse)] for 1 h at RT. After washing three times for 30 min with TBST at RT, the signal was visualized using HRP Substrate solution (Serva, 42582.01). For autoradiography, either hypersensitive X-ray films (CL-XPosure Film, Thermo Fischer Scientific, 34090) (exposure was done at −80°C using low energy screen) or a phosphorimager scanner (GE Healthcare, Typhoon) was used to detect the ^35^S Methionine-labelled proteins.

### 4.16. Chromatin preparation

About 120 ovaries of adult females of 2–5 days were dissected from each genotype in Schneider’s *Drosophila* medium. To avoid the induction of stress responses, the tissues were immediately fixed within 5 min after dissection with 1% freshly prepared PFA-PBS for 10 min at RT with rotation. Fixation was quenched by adding 125 mM Glycine from freshly prepared 2.5 M stock solution for 5 min at RT with rotation. Then, the ovaries were washed three times in 1 × PBS and two times in Farnham buffer (5 mM HEPES pH 8.0, 85 mM KCl, 0.5% NP-40, 1 × protease inhibitor cocktail) for 5–5 min each at RT with rotation. Before the second wash with Farnham buffer, the ovaries were separated to ovarioles by pipetting them up and down. Then, the ovarioles were collected by centrifugation (4000 × *g*, 1 min, 4°C). After discarding the supernatant, the ovarioles were resuspended in 250 µl of RIPA buffer (20 mM Tris pH 7.4, 150 mM NaCl, 1% NP-40, 0.5% Sodium Deoxycholate, 0.1% SDS, 1 × protease inhibitor cocktail), transferred to a douncer and carefully dounced until the solution became milky (around 20 strokes). The samples were incubated on ice for 10 min, transferred to sonication tubes and sonicated for 10 min with the following settings: peak power 75.0, duty factor 10.0, cycles/burst 200 (Covaris M220 Focused-ultrasonicator). The sonicated samples were centrifuged at 18 000 × *g* for 15 min at 4°C, and the supernatant was used for ChIP experiments.

### 4.17. Chromatin immunoprecipitation

In the ChIP experiments, the magnetic beads (Dynabeads Protein A, 30 mg ml^−1^, Invitrogen, 10 002D) were first washed three times with 5 mg ml^−1^ freshly prepared and filtered BSA (10 µl of beads for each pre-clearing and 50 + 50 µl for each IPs), and then 10 µl of clean beads were added to each chromatin lysate to pre-clear the samples. After rotation for 2 h at 4°C, 5% of the pre-cleared chromatin was saved for input fraction, and the remaining clean beads were divided into 50 µl aliquots. After adding 1–1 ml of BSA to the samples, the antibody was added to one tube (IP) and a second tube was saved for NAC. Samples were then incubated for 2 h at 4°C on a rotator to conjugate the antibody. After washing 1 × with BSA, equal volume of the pre-cleared chromatin was added to each sample and incubated for 4 h at 4°C. The beads were washed after the IP five times with 1 ml of LiCl buffer (10 mM Tris pH 7.5, 500 mM LiCl, 1% NP-40, 1% sodium deoxycholate) with rotation and once with 1 ml of TE buffer without rotation. Next, 200 µl of Proteinase K buffer (200 mM Tris pH 7.4, 25 mM EDTA, 300 mM NaCl, 2% SDS) and 100 µg Proteinase K (Thermo Scientific, EO0491) were added, and the samples were incubated for 2–3 h at 55°C and then overnight at 65°C to reverse cross-link. For the input fractions, first 1 µl RNase A (from 10 mg ml^−1^ stock) was added for 1 h at 37°C, then 200 µl of Proteinase K buffer, 1 mg ml^−1^ Proteinase K and 300 mM NaCl was added and the mixture was incubated for 2–3 h at 55°C and then overnight at 65°C. The DNA was extracted with the phenol-chloroform method, and DNA pellets were resuspended in 40 µl of DNase- and RNase-free water (Lonza, AccuGENE, BE51200); 1 µl of DNA was used as template in the qPCR reactions.

### 4.18. Total RNA isolation, qPCR and statistical analysis

In these experiments, the protocol described in [78] was followed. In brief, five pairs of ovaries were dissected from 2 to 5 d old females in Schneider’s *Drosophila* medium. The ovaries were carefully washed with 1 × PBS then total RNA isolation was performed using TRIzol reagent (Invitrogen, 15596–018), and RNA pellets were resuspended in 30 µl of DNase- and RNase-free water; 1.5 µl of RNA was used as template in the qPCR reactions.

The qPCR reactions were carried out according to the manufacturer’s instructions (Bioline, SensiFAST SYBR Hi-ROX Kit, BIO-92005) in a volume of 20 µl. Primers are listed in table 1.

Shapiro–Wilk’s test was used to test for normality of data distribution. Student’s *t*-tests were performed for pairwise comparisons. Samples were compared pairwise by Student’s *t*‐test. Graphs were created with GraphPad Prism 9.1.2 (GraphPad Software). Statistical significance is marked with ***p < 0.0001, **p < 0.001, *p < 0.05 and n.s. (not significant): *p* > 0.05.

## Data Availability

The authors declare that all data are included in the manuscript and supplementary information [79]. Further inquiries can be directed to the corresponding authors.
